# CHA_2_DS_2_-VASc Score as a Predictor for Left Atrial Thrombus or Spontaneous Echo Contrast in Patients with Nonvalvular Atrial Fibrillation: A Meta-Analysis

**DOI:** 10.1155/2020/2679539

**Published:** 2020-07-11

**Authors:** Ping Sun, Zhi Hao Guo, Hong Bin Zhang

**Affiliations:** Vasculocardiology Deparment, Cangzhou Central Hospital, No. 16 Xinhua Road, Yunhe Qu, Cangzhou 061000, China

## Abstract

**Objective:**

This meta-analysis aimed at exploring the predictive value of CHA_2_DS_2_-VASc score for the left atrial thrombus (LAT) or left atrial spontaneous echo contrast (LASEC) in patients with nonvalvular atrial fibrillation (NVAF).

**Methods:**

PubMed, Embase, Web of Science, ScienceDirect, Cochrane Library, and Chinese core journals of the CNKI and Wanfang databases were searched to identify all the relevant papers that were published up to January 2020. The data were extracted for pooled odds ratios (ORs) with 95% confidence intervals (CIs), heterogeneity, subgroup, publication bias, and sensitivity analysis.

**Results:**

Overall, 15 studies containing 6223 patients with NVAF were enrolled. All studies were evaluated for LAT, and 12 studies were evaluated for LASEC. The pooled analysis using a random-effects model showed that a high CHA_2_DS_2_-VASc score was related with LAT/LASEC (pooled OR = 1.59, 95% CI: 1.35–1.88, *P* < 0.001) with high heterogeneity (*I*^2^ = 76.9%, *P* < 0.001) and LAT (pooled OR = 1.83, 95% CI: 1.44–2.33, *P* < 0.001) with high heterogeneity (*I*^2^ = 79.4%, *P* < 0.001). The subgroup analysis demonstrated that the sample size may be the main source of heterogeneity. Although the Begg's funnel plot based on 15 studies for LAT/LASEC (*P* = 0.029) and 12 studies for LAT (*P* = 0.046) indicated the presence of publication bias among the included studies, the trim-and-fill method verified the stability of the pooled outcomes. In addition, sensitivity analysis indicated that all effects were stable.

**Conclusion:**

The results of this meta-analysis showed that the CHA_2_DS_2_-VASc score is related with LAT and LASEC in patients with NVAF. However, more studies are warranted to address this issue.

## 1. Introduction

Atrial fibrillation (AF) is a fairly common arrhythmia worldwide, and 33 million people are estimated to be suffering from this condition [1]. Worryingly, the left atrial thrombus (LAT) and left atrial spontaneous echo contrast (LASEC) formation, which are among the most frequent complications that develop in patients with AF, are related to high rates of stroke and mortality [2–4]. The transesophageal echocardiography (TEE) is considered the global standard for detecting LAT with 97% sensitivity and 100% specificity [5, 6]. However, TEE facilities are limited in developing countries and depend greatly on the operator's skill and experience. Thus, the clinical methods for LAT/LASEC prediction and risk assessment in a timely manner are particularly important.

CHA_2_DS_2_-VASc is a simple, clinical risk factor-based approach to thromboprophylaxis. Although the CHA2DS_2_-VASc score has been widely used to evaluate the risk recurrence of AF [7] and ischemic stroke in patients with AF [8], there has been limited evidence of LAT/LASEC prediction according to CHA2DS2-VASc scores. In 2015, a letter to an editor [9] reported that a high CHA_2_DS_2_-VASc score can predict LAT/LASEC by analyzing six relevant studies [10–15]. Nevertheless, several subsequent studies [16–25] are not included in this meta-analysis, and these studies have shown inconsistent conclusions. Therefore, an updated meta-analysis was performed to evaluate the association between CHA_2_DS_2_-VASc score and the risk of LAT or LASEC, and the discrimination ability of CHA_2_DS_2_-VASc score was further determined for the prediction of LAT/LASEC.

## 2. Materials and Methods

This meta-analysis was based on the preferred reporting items for the systematic review and meta-analysis (PRISMA) project [26]. All data were collected from published trials. Hence, an additional ethical approval was not necessary.

### 2.1. Search Strategy

PubMed, Web of Science, ScienceDirect, Cochrane Library, and the Chinese core journals of the CNKI and Wanfang database were systematically searched to identify relevant studies from inception to January 2020 by using the following search terms: “CHA_2_DS_2_-VASc,” “thrombus,” and “fibrillation.” The reference lists of some major articles and reviews were manually checked to avoid missing relevant studies.

### 2.2. Inclusion and Exclusion Criteria

Studies were included if they satisfied the following criteria: original clinical trial, studies that reported the relation between CHA_2_DS_2_-VASc score and LAT/LASEC in patients with AF, and sufficient information to evaluate odds ratios (ORs) with 95% confidence intervals (CIs). Case reports, reviews, conference papers, editorials, and animal studies were excluded. The most informative study was included for the repeated studies carried out among identical research populations. Studies were identified using the above search strategy by two independent reviewers. A third reviewer was consulted when faced with uncertainty regarding eligibility.

### 2.3. Data Collection

Two reviewers extracted data concerning patient characteristics and clinical outcomes by using a standard data collection form. The first author's name, year of publication, study design, region, paroxysmal AF percentage, age, ample size, number of LAT and LASEC, and CHA_2_DS_2_-VASc score were collected from each study that met the inclusion criteria.

### 2.4. Quality Assessment of the Selected Articles

The quality of the included studies was separately assessed by two reviewers by using the Quality Assessment of Diagnostic Accuracy Studies-2 (QUADAS-2) tool [27]. All eligible studies were evaluated on the basis of four domains: patient selection, index test, reference standard, and flow and timing. The ratings were cross-checked, and the difference was solved by a third reviewer.

### 2.5. Statistical Analysis

The STATA 14.0 (Stata-Corp LP, College Station, TX, USA) was applied to assess statistical significance. The summary effect size was expressed as an OR with corresponding 95% CI. Heterogeneity between studies was assessed using the Cochran *χ*^2^-based Q-statistic and *I*^2^ test. The *I*^2^ values of 25%, 50%, and 75% indicated low, moderate, and high levels of heterogeneity, respectively. The fixed-effect model was used if *P* > 0.1 and *I*^2^ < 50%. Otherwise, the random-effects model was used. If heterogeneity exists, the possible reasons were investigated and reported through subgroup analysis. Furthermore, sensitivity analysis was performed to evaluate the robustness of the meta-analysis results. Begg's funnel plot was used to explore publication bias, and the trim-and-fill method was used to adjust the effect of publication bias if present (*P* < 0.05). A two-sided *P* < 0.05 was considered statistically significant.

## 3. Results

### 3.1. Search Strategy

**Figure 1:**
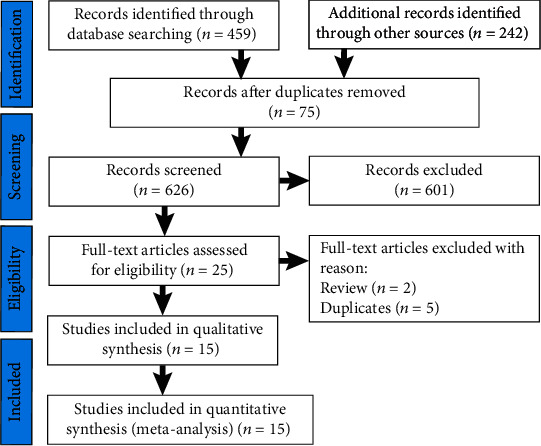
Flow diagram of the study screening and selection process.

Figure 1 displays the literature identification and selection process. The electronic searches yielded 701 potentially relevant studies. Among these studies, 676 were excluded due to duplication or irrelevance to the topic. The remaining 25 studies underwent a full-text review, and 15 studies [10–25] were finally included. The other 10 studies were excluded because 5 repeated studies were carried out among identical research populations; 3 studies were unable to extract related data; and the other 2 were reviews.

### 3.2. Characteristics and Quality of Included Studies

**Figure 2:**
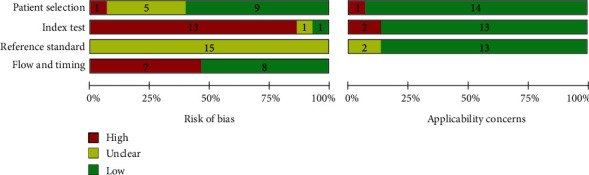
Risk of bias summary of the included studies.

The baseline characteristics of fifteen eligible studies in the meta-analysis are summarized in Table 1. The mean age of the participants was 59–70 years. The sample size of studies ranged from 64 to 1359 with a mean sample size of 415. Among the 15 studies [10–25] included in the final meta-analysis, 7 were from the China [10, 13, 16, 17, 21, 23, 25], 2 were from Poland [18, 20], 2 were from USA [22, 25], 2 were from Japan [12, 19], 1 was from Greece [11], and 1 was from Turkey [14]. The percentage of paroxysmal AF varied from 24.1% to 88%. The evaluations of the risk of bias and applicability concerns by using the QUADAS-2 are shown in Figure 2 and Figure S1. The final assessment results consider the quality and risk of bias of each study acceptable.

### 3.3. Effect Sizes

**Figure 3:**
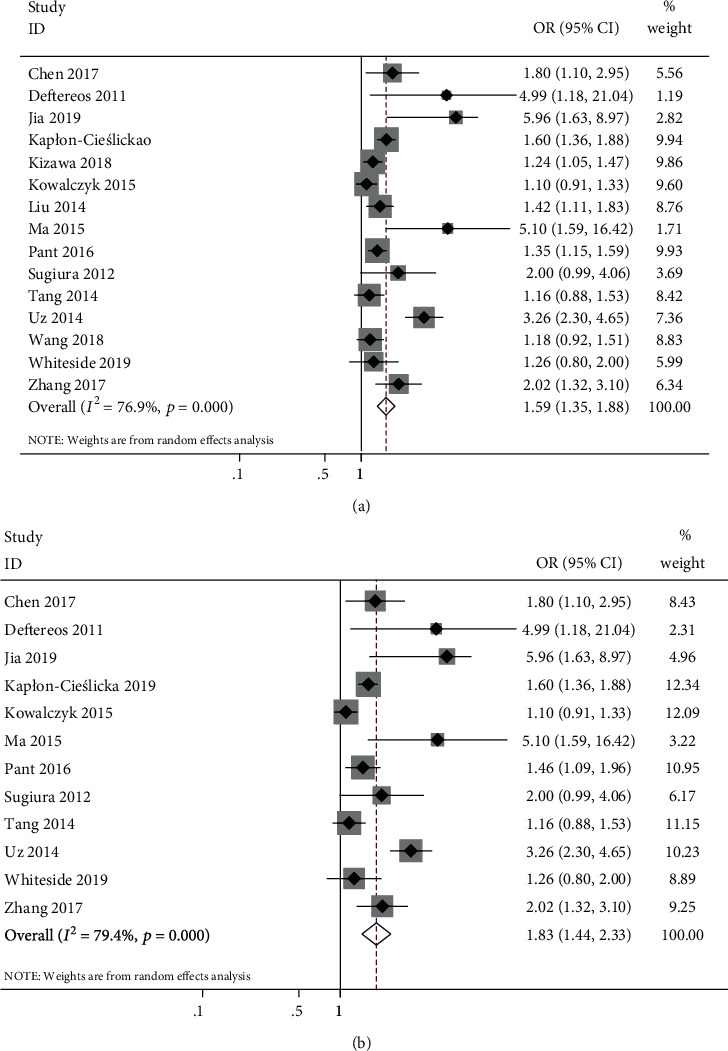
Forest plot of the association between CHA_2_DS_2_-VASc score and LAT/LASEC. (a) LAT/LASEC. (b) LAT. LAT: left atrial thrombus; LASEC: left atrial spontaneous echo contrast.

The data from the different studies had a statistical heterogeneity. Therefore, the random-effects model was used for data analysis. The pooled analysis showed that a high CHA_2_DS_2_-VASc score was related with LAT/LASEC (pooled OR = 1.59, 95% CI: 1.35–1.88, *P* < 0.001; *I*^2^ = 76.9%, *P* < 0.001; Figure 3(a)) based on the 15 included studies [10–25] and related with LAT (pooled OR = 1.83, 95% CI: 1.44–2.33, *P* < 0.001; *I*^2^ = 79.4%, *P* < 0.001; Figure 3(b)) based on the 12 included studies [11–14, 16–18, 20–22, 24, 25].

### 3.4. Subgroup Analysis

Subgroup analysis were conducted to investigate the source of heterogeneity (Table 2). The subgroup analysis for LAT/LASEC with 15 studies [10–25] was performed on the basis of sample size (≥415 vs. <415), publication year (before 2015 vs. after 2015), region (Asia vs. non-Asia), and proportion of male (≥65.3% vs. <65.3%). Sample size may account for the source of heterogeneity.

The subgroup analysis for LAT with 12 studies [11–14, 16–18, 20–22, 24, 25] was performed on the basis of sample size (≥379 vs. <379), publication year (before 2015 vs. after 2015), region (Asia vs. non-Asia), and proportion of male (≥66.0% vs. <66.0%). However, these factors did not account for the source of heterogeneity (Table 2).

### 3.5. Publication Bias Assessment

**Figure 4:**
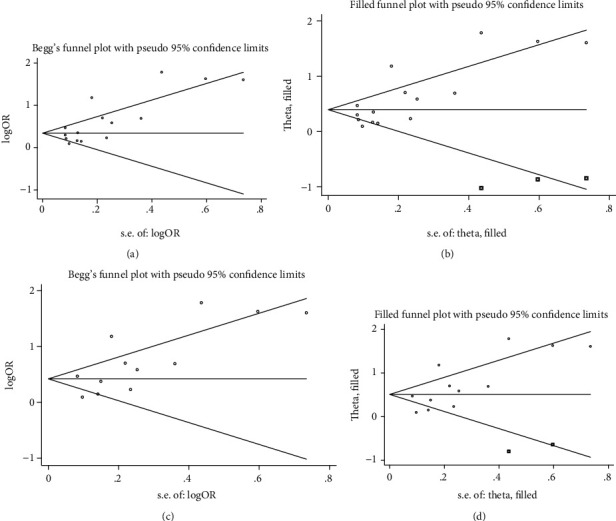
Funnel plot to test for publication bias. (a) Begg's test and (b) trim-and-fill method for LAT/LASEC; (c) Begg's test and (d) trim-and-fill method for LAT.

The publication bias for LAT/LASEC in 15 studies [10–25] was detected using the Begg's test (*P* = 0.029) (Figure 4(a)). The trim-and-fill results show that three necessary studies have been missed. The adjusted fixed-effects pooled OR of 1.48 (95% CI: 1.24–2.13, *P* < 0.001) calculated using the trim-and-fill method was consistent with the original analysis (OR = 1.59, 95% CI: 1.35–1.88, *P* < 0.001; Figure 4(b)).

The publication bias for LAT in 12 studies [11–14, 16–18, 20–22, 24, 25] was detected using Begg's test (*P* = 0.064) (Figure 4(c)). The trim-and-fill results show that two necessary studies have been missed. The adjusted fixed-effects pooled OR of 1.66 (95% CI: 1.30–2.13, *P* < 0.001) calculated using the trim-and-fill method was consistent with the original analysis (OR = 1.83, 95% CI: 1.44–2.33, *P* < 0.001; Figure 3(d)).

### 3.6. Sensitivity Analysis

**Figure 5:**
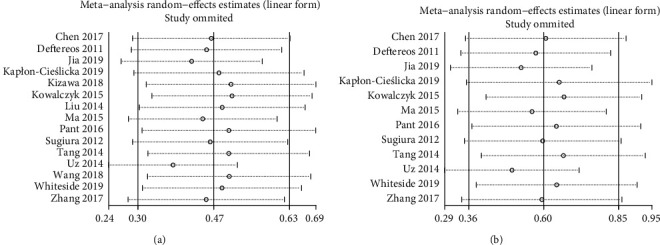
Sensitivity analysis of the correlation between CHA_2_DS_2_-VASc score and LAT/LASEC. (a) LAT/LASEC. (b) LAT. LAT: left atrial thrombus; LASEC: left atrial spontaneous echo contrast.

Sensitivity analysis showed no noticeable change in the statistical significance of all outcomes by removing the single studies. This finding indicated that all effects were stable (15 LAT/LASEC-based studies, Figure 5(a); 12 LAT-based studies, Figure 5(b)).

## 4. Discussion

This meta-analysis and systematic review provided further viewpoint on the relationship between the CHA_2_DS_2_-VASc score and LAT/LASEC in patients with AF, and the results showed that patients with high CHA_2_DS_2_-VASc score had 1.59- and 1.83-fold higher risks of LAT/LASEC and LAT, respectively. This result was consistent with that of a previous study.

A previous study reported that the pooled analysis in the random-effects model demonstrated a statistically significant 70% increase in the detection of LAT/LASEC (OR = 1.70; 95% CI: 1.16–2.48) and 122% increased risk for detecting LAT (OR = 2.22; 95% CI: 1.11–4.44) from higher CHA_2_DS_2_-VASc score to lower CHA_2_DS_2_-VASc score based on the four studies [9]. This finding was consistent with our result. The strength of our results came from the analysis of a large number of patients and the high quality of data obtained, which was confirmed by the relationship between the CHA2DS_2_-VASc and the LAT/LASEC.

Notably, predicting the LAT/LASEC in AF with CHA_2_DS_2_-VASc score remains challenging. Some studies have indicated that patients who are categorized as low risk by the CHA_2_DS_2_-VASc score (i.e., score 0 in males or 1 in females) also have a risk of LAT/LASEC [13, 28–30]. In other words, the CHA_2_DS_2_-VASc score needs to be improved and perfected. Van Chien et al. has increased the predictive ability (*χ*^2^) from 3.53 to 33.48 by adding the left atrial volume index and the left atrial negative strain rate in the two-chamber view to the CHA_2_DS_2_-VASc score [31]. Similarly, predictive ability has increased by 13% when the left atrial emptying fraction is added to the CHA_2_DS_2_-VASc score, as shown in Kim et al.'s study [32]. In addition, the areas under the curve have increased from 0.70 to 0.81 by adding the renal dysfunction and the AF type to the CHA_2_DS_2_-VASc score, as shown in Kapłon-Cieślicka et al.'s study [18]. New studies may be necessary to test if the addition of items, such as left atrial volume index, left atrial negative strain rate in two-chamber view, renal dysfunction, and AF type, to the CHA_2_DS_2_-VASc can increase the diagnostic efficiency of the score on LAT/LASEC.

This review has several important limitations that need to be acknowledged. First, the eligible articles included in our meta-analysis were restricted to studies published in English and Chinese and likely caused selection bias. Second, complete information regarding some variables, such as renal dysfunction, AF type, and TEE, were lacking in the included article. Third, heterogeneity among studies existed and our analysis should be interpreted with caution.

In conclusion, our meta-analysis suggested that CHA_2_DS_2_-VASc score is a valuable predictor for LAT/LASEC in patients with AF. However, further well-designed studies are still warranted to confirm our findings.

## Figures and Tables

**Table 1:** Study characteristics.

First author (year)	Design	Region	Paroxysmal AF (%)	Age	Male (%)	Warfarin (%)	*N*	LAT, *n* (%)	LAT		LAT/LASEC, *n* (%)	LAT/LASEC	
									Present	Absent		Present	Absent
Chen 2017	P	China	59.3%	59.4 ± 11.8	61.9%	42.9%	189	13 (6.87)	2.31 ± 1.32	1.29 ± 1.28	NA	NA	NA
Deftereos 2011	R	Greece	NA	62.2 ± 1.2	65.1%	NA	86	15 (17.44)	3.8 ± 0.3	1.3 ± 0.1	NA	NA	NA
Jia 2019	P	China	82%	59 ± 13	66.2%	NA	397	38 (9.57)	NA	NA	NA	NA	NA
Kapłon-Cieślicka 2019	R	Poland	61%	60 (53-66)	66%	NA	1033	59 (5.71)	3 (2-4)	2 (1-3)	NA	NA	NA
Kizawa 2018	R	Japan	62%	66 ± 10	69%	22%	581	NA	NA	NA	147 (25.30)	3.1 ± 1.5	2.0 ± 1.4
Kowalczyk 2015	R	Poland	NA	64 ± 8.8	64%	NA	64	30 (46.85)	3.2 ± 1.6	2.80 ± 1.8	NA	NA	NA
Liu 2014	R	China	83%	60.8 ± 11.2	64.2%	NA	525	NA	NA	NA	57	2.77 ± 1.12	2.33 ± 1.17
Ma 2015	R	China	88.0%	61.12	57.3%	NA	164	32 (19.51)	3.4 ± 1.8	1.9 ± 1.4	NA	NA	NA
Pant 2016	P	USA	26%	65 ± 12	70%	48%	261	17 (6.51)	4.4 ± 1.6	3.0 ± 1.8	85 (31.42)	NA	NA
Sugiura 2012	P	Japan	66.2%	62 ± 11	77%	100%	225	23 (10.22)	3 ± 2	2 ± 1	NA	NA	NA
Tang 2014	R	China	66.6%	58.1 ± 11.2	71.0%	12.7%	1359	61 (4.49)	NA	NA	NA	NA	NA
Uz 2014	R	Turkey	NA	70.1 ± 9.8	49%	32%	309	32 (10.36)	NA	NA	70 (22.65)	NA	NA
Wang 2018	R	China	28.6%	66.1 ± 10.8	57.4%	39.6%	472	NA	NA	NA	80 (16.95)	3.79 ± 1.75	2.65 ± 1.76
Whiteside 2019	R	USA	24.1%	65.8 ± 11.9	61.5%	0%	226	7 (3.10)	3.43 ± 1.40	2.81 ± 1.62	NA	NA	NA
Zhang 2017	R	China	12.9%	67.62 ± 6.96	57.3%	NA	332	116 (50)	2.88 ± 1.51	1.50 ± 1.22	NA	NA	NA

Continuous variables are presented as mean, mean ± standard deviation, or median (interquartile range), and categorical variable is presented as no. (%).

**Table 2:** Subgroup analysis of potential sources of heterogeneity.

	Heterogeneity factors		No. of studies	OR (95% CI)	*P* value	*I* ^2^ (*P* value)
For LAT/LASEC with 15 studies	Sample size	≥415	5	1.33 (1.17–1.52)	<0.001	47.9% (0.104)
		<415	10	2.02 (1.48–2.75)	<0.001	82.4% (<0.001)
	Publication year	Before 2015	7	1.83 (1.26–2.66)	0.002	84.5% (<0.001)
		After 2015	8	1.49 (1.26–1.77)	<0.001	66.7% (0.003)
	Region	Asia	10	1.81 (1.39–2.35)	<0.001	81.8% (<0.001)
		Non-Asia	5	1.36 (1.12–1.66)	0.002	66.4% (0.018)
	Proportion of male	≥65.3%	9	1.42 (1.20–1.67)	<0.001	70.4% (0.001)
		<65.3%	6	1.93 (1.28–2.91)	0.002	81.6% (<0.001)
For LAT with 12 studies	Sample size	≥379	3	1.81 (1.11–2.97)	<0.001	85.6% (0.001)
		<379	9	1.89 (1.37–2.62)	0.018	79.7% (<0.001)
	Publication year	Before 2015	6	2.03 (1.24–3.32)	0.005	87.1% (<0.001)
		After 2015	6	1.73 (1.37–2.19)	<0.001	57.6% (0.038)
	Region	Asia	7	2.37 (1.54–3.64)	<0.001	80.7% (<0.001)
		Non-Asia	5	1.39 (1.11–1.76)	0.005	66.8% (0.017)
	Proportion of male	≥66.0%	6	1.51 (1.17–1.96)	0.002	78.0% (<0.001)
		<66.0%	6	2.28 (1.54–3.38)	<0.001	65.5% (0.013)

CI: confidence intervals; SE: standard error.

## Data Availability

The data used to support the findings of this study are included within the article.

## Abbreviations

AF: Atrial fibrillation
AVAF: Nonvalvular atrial fibrillation
LAT: Left atrial thrombus
LAEAS: Left atrial spontaneous echo contrast
TEE: Transesophageal echocardiography
ROC: Receiver operating characteristic.

## Supplementary Materials

Supplementary Materials Supplementary figure S1: risk of bias of the included studies.
